# Healthy People and Interested Students: Medical and Pharmacy Students’ Knowledge and Attitudes Regarding Public Health

**DOI:** 10.3390/pharmacy9040176

**Published:** 2021-10-28

**Authors:** Richard P. Boyd, Trate A. DeVolld, Natalie A. DiPietro Mager, William J. Burke

**Affiliations:** 1 Ohio University Heritage College of Osteopathic Medicine, 6775 Bobcat Way, Dublin, OH 43016, USA; rb141919@ohio.edu (R.P.B.); burke@ohio.edu (W.J.B.); 2 Department of Pharmacy Practice, Ohio Northern University Raabe College of Pharmacy, 525 S. Main St., Ada, OH 45810, USA; t-devolld@onu.edu; 3 Association for Prevention Teaching and Research Healthy People Curriculum Task Force, 1001 Connecticut Avenue, NW Suite 610, Washington, DC 20036, USA

**Keywords:** public health, healthy people programs, preventive health services, students, health occupations, students, medical, students, pharmacy

## Abstract

Little is known about health professions students’ awareness and attitudes regarding public health in the United States. Therefore, the purpose of this study was to assess medical and pharmacy students’ knowledge and interest in the Healthy People initiative as well as perceptions of public health content in their curricula. An electronic survey was distributed in March 2021 in seven schools across Ohio; participation was incentivized through a USD 5 donation to the Ohio Association of Foodbanks to aid in COVID-19 relief efforts (maximum USD 1000) for each completed survey. A total of 182 medical students and 233 pharmacy students participated (12% response rate). Less than one-third of respondents reported familiarity with Healthy People and correctly identified the latest edition. However, nearly all respondents agreed public health initiatives are valuable to the American healthcare system. Almost all students expressed a desire to practice interprofessionally to attain public health goals. Both medical and pharmacy students recognized core public health topics in their curricula, and nearly 90% wanted more information. These findings indicate that the majority of medical and pharmacy students in Ohio believe public health initiatives to be important, yet knowledge gaps exist regarding Healthy People. This information can guide curricular efforts and inform future studies of health professions students.

## 1. Introduction

Although the United States (U.S.) spends more on health care per capita than any other high-income country, it reports the highest rates of infant mortality and lowest life expectancy among them. The percentage of the American adult population that is obese or has multiple chronic conditions is also higher than comparable countries [1]. Additionally, the U.S. struggles with significant health disparities based on race, ethnicity, socioeconomic status, and geography [2].

The national Healthy People initiative seeks to improve health outcomes by providing a foundation for health and health promotion goals and aiming to eliminate health disparities. Healthy People is coordinated by the U.S. Department of Health and Human Services Office of Disease Prevention and Health Promotion. At the start of each decade, measurable objectives that promote health and reduce the burden of disease are published with the intent for public health and clinical health professionals to work toward those targets over the next 10 years. This future-oriented approach to improving the nation’s health and well-being began in 1979; the fifth iteration, Healthy People 2030, was released in August 2020 [3].

Healthy People provides direction for public health action by identifying the nation’s most pressing health needs. Table 1 lists the blueprint for Healthy People 2030. The latest version of Healthy People allows users of the website to set local targets for populations in their own communities based on national data [3]. As “micro” interventions in individual patients and practice settings can result in significant improvement at the “macro” level for populations, clinical health professionals play an impactful role in meeting Healthy People objectives [4]. Moreover, interprofessional collaborations among these clinical health professionals are key to reducing fragmented care, lowering health care costs, and improving health outcomes; collaborative practice agreements (CPA) between physicians and pharmacists are one such example [5,6]. Ongoing federal and state initiatives to grant pharmacists provider status and remove administrative barriers for reimbursement for clinical services can further facilitate CPA models [7,8]. 

Therefore, it is important that health professions students receive adequate education and training regarding Healthy People through their professional school curricula to enable them to affect meaningful change in their future practices. To that end, the Association for Prevention Teaching and Research convened the Healthy People Curriculum Task Force (HPCTF) in 2002, a group that currently represents eight health professional education associations, including the Association of American Medical Colleges, the American Association of Colleges of Osteopathic Medicine, and the American Association of Colleges of Pharmacy [9]. The HPCTF maintains the Clinical Prevention and Population Health (CPPH) curriculum framework that suggests topics encompassing public health (i.e., protecting the health of populations) and population health (i.e., advancing health outcomes of groups of individuals) to be included in health professions education [10,11]. Health professions students must understand these distinct but intertwined areas in order to effectively provide individual- and population-oriented prevention and health promotion services [10].

To date, no studies have assessed health professions students’ awareness of Healthy People 2030 or have examined perceptions of their professional curricula in regard to instruction on the key elements of the CPPH framework. Therefore, the primary objective of this study was to assess medical and pharmacy students’ knowledge of the federal Healthy People initiative and interest to affect its priority areas. The secondary objective was to assess students’ perception of public health content in their curricula and whether they would like more information regarding public health.

## 2. Materials and Methods

An electronic survey using Qualtrics software (Provo, UT) was developed to collect data from medical and pharmacy students in order to determine whether they (1) are knowledgeable of the Healthy People initiative; (2) perceive learning about certain aspects from the CPPH in their professional curricula; (3) want additional education on public health and preferred methods of instruction; (4) share common public health goals from Healthy People 2030; (5) anticipate barriers in addressing health promotion with patients; and (6) have interest in interprofessional collaboration. Selected demographic characteristics of respondents, including anticipated graduation date, were also captured. 

The final surveys consisted of 35 questions for medical students and 36 questions for pharmacy students. The two surveys asked the same questions, with one additional question for the pharmacy cohort assessing interest in provider status. Question types included Likert scale, ranking, and multiple choice. The questions assessing the anticipated barriers to health promotion and related services as well as students’ preferred methods of instruction for public health content listed possible answers in a multiple-choice format and allowed for a free text response if students felt their ideas were not represented. The study was deemed exempt by the Ohio University Institutional Review Board (IRB) with the Ohio Northern University IRB agreeing that the Ohio University IRB may serve as the IRB of Record.

Prior to distribution, the survey was pre-tested to obtain feedback regarding questionnaire design and potential computer-based or technical problems by a purposive sample of medical and pharmacy residents as well as medical and pharmacy students outside of the state of Ohio who did not qualify to take the survey. Pre-test participants reported little to no survey fatigue, that instructions and questions were clear, and that they experienced no technical difficulties; therefore, no changes needed to be made to the survey.

A link to the survey was sent via email in March 2021 to students enrolled at two schools of medicine (one allopathic, one osteopathic) and five schools of pharmacy in Ohio. The email advised the student that the survey was voluntary and anonymous. To minimize potential participant bias, the email did not disclose that the focus of the survey was Healthy People and public health, but instead referred to “educational experiences and future practice plans” as the survey topic. Participation was incentivized through a $5 donation to the Ohio Association of Foodbanks to aid in COVID-19 relief efforts for each completed survey up to a maximum of $1000 due to funding constraints. Informed consent was obtained from participants via the first question of the survey. The survey remained open for 21 days, with one reminder email sent within this timeframe. 

Data were analyzed using Microsoft Office Excel 2019 (Redmond, WA, USA) and IBM SPSS 25 (Armonk, NY, USA) software. Descriptive statistics were used to calculate frequencies of responses, and chi-square or Kruskal–Wallis tests were used as appropriate to identify potential differences in responses between medical and pharmacy students and between students’ year of study. Alpha was set a priori at 0.05, with the Bonferroni correction applied to the Kruskal–Wallis tests.

## 3. Results

A total of 182 medical students and 233 pharmacy students responded to the survey out of an estimated 3590 students who were invited to participate (12% response rate). The difference in response rate between medical students (10%) and pharmacy students (13%) was statistically significant (*p* = 0.019). Eighty-one percent of the surveys were completed in their entirety. As no questions in the survey were marked as mandatory, 19% of respondents left at least one question unanswered; therefore, reported percentages are calculated from the total number of respondents who answered each question. 

Table 2 lists selected demographic information for respondents. Data from the six survey focus areas are grouped for discussion below into two main categories: findings related to students’ knowledge of Healthy People and perceptions of public health content in their curricula (Table 3) and findings related to students’ interest and ideas for future practice (Table 4).

## 4. Knowledge of Healthy People and Perceptions of Curricula

### 4.1. Awareness of Healthy People Initiative

Overall, less than one-third of all respondents reported that they were familiar with the Healthy People initiative, but pharmacy students were twice as likely as medical students to claim familiarity (39% vs. 17%, *p* < 0.001). Pharmacy students also more often reported learning about Healthy People in their professional school curricula (36% vs. 7%, *p* < 0.001). Over 90% of students were able to correctly identify the mission of Healthy People, but less than 30% could identify the current edition. There were no statistically significant differences in responses to these questions based on student year of study.

### 4.2. Perceived Inclusion of the CPPH in Professional Curricula

More than 70% of medical and pharmacy students agreed that they felt knowledgeable about public health-related topics. The majority of respondents either strongly or somewhat agreed that their curriculum included the four primary components of the CPPH framework (foundations of public health; clinical preventive services and health promotion; clinical practice and population health; and health systems and health policy). Survey participants also reported learning about the seven topic areas included in the Healthy People 2020 objective for health professions education (counseling for disease prevention and health promotion; cultural diversity; social determinants of health; evaluation of health sciences literature; public health systems; environmental health; global health), consistent with published end-of-decade data [12].

### 4.3. Desire for Additional Education on Public Health and Preferred Methods of Instruction

Respondents from both groups expressed a desire for more information about public health than their curriculum provided, with nearly 90% of medical and pharmacy students expressing at least some interest. Although there was no significant difference in wanting more information between the groups (*p* = 0.164), a higher percentage of medical students strongly desired more public health inclusion in their curriculum than pharmacy students (46% vs. 38%). The preferred methods for the delivery of additional information from both groups were elective courses (59%), lecture or series of lectures as part of an existing class (51%), continuing education in future practice (44%), service learning (44%), experiential learning (41%), or interprofessional exercises (32%). Least popular among students was self-directed learning (17%).

## 5. Interest and Ideas for Future Practice

### 5.1. Common Public Health Goals from Healthy People 2030

**Figure 1:**
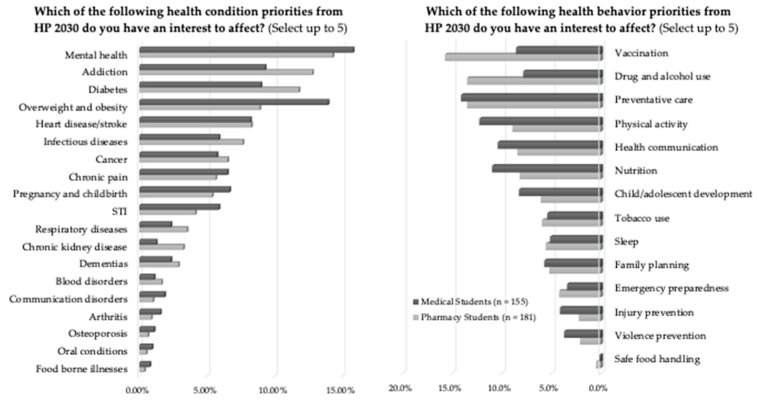
Medical and pharmacy students’ interest to affect Healthy People 2030 priorities.

Ninety-eight percent of respondents agreed that public health initiatives are valuable to the American health care system. Both groups of students exhibited interest in affecting similar priorities from Healthy People 2030 in future practice (Figure 1). Among the five Healthy People 2030 focus areas, a higher percentage of pharmacy students ranked health conditions as most significant (*p* < 0.001). Medical students placed more significance on settings and systems and social determinants of health (*p* < 0.001). When asked specifically which determinants they thought they could impact in future practice, the most common response from both groups was health care access and quality (86%), followed by social and community support (64%), education (58%), neighborhood and built environments (50%), and economic stability (38%).

### 5.2. Anticipated Barriers in Addressing Healthy People Goals

The majority of respondents (65%) acknowledged that there would be barriers to providing health promotion and services outlined by the Healthy People initiative in their future practices. Lack of time (71%), lack of coordination with other health care providers (63%), lack of personnel and resources (60%), and lack of reimbursement (49%) were chosen as the top issues. Some students also provided free-text responses, such as “lack of universal health insurance” and “lack of patient resources (time, money, etc.) to be able to make change”. Another student pointed out that implementing certain services could be a challenge due to a lack of “government or institutional support”. Interestingly, only a small percentage of students reported lack of relevance to area of practice (12%) as an anticipated barrier. Similarly, lack of knowledge or clinical skills was not often anticipated as a barrier among respondents (17%). 

### 5.3. Interest in Interprofessional Collaboration

When asked about the importance of working with health care providers in other professions to achieve public health goals, both groups placed almost unanimous support behind this concept. Interest in forming collaborative partnerships with each other was almost as high, with over 80% of both groups answering “strongly agree” to interest in such an opportunity. Furthermore, 96% of the pharmacy student respondents expressed interest in acquiring provider status. As a significantly higher percentage of pharmacy students than medical students selected lack of reimbursement as an anticipated barrier (57% vs. 42%, *p* = 0.007), obtaining provider status could help to overcome that obstacle.

## 6. Discussion

These findings add to the limited literature about health professions students’ knowledge and attitudes regarding Healthy People and public health goals. A majority of medical and pharmacy students surveyed believe public health initiatives are important but reported gaps in knowledge related to Healthy People. The results of this study were consistent with previous studies with emergency medicine physician residents and pharmacy residents, where over 80% and 40%, respectively, reported unfamiliarity with the Healthy People initiative [13,14]. 

Although actual curricular content was not assessed but rather student perceptions or recollections of content, these findings do imply that additional efforts should be made to increase awareness or retention of information regarding Healthy People among medical and pharmacy students. It is important to educate health professions students about Healthy People because, by working towards these objectives, the health of populations, resilience to public health threats such as the COVID-19 pandemic, and health disparities can be improved [3]. Faculty should refer to the CPPH curriculum framework as well as their discipline’s accreditation standards to ensure they are adequately preparing their students [10]. 

Given that few respondents identified a lack of relevance or clinical skills as potential barriers to implementing Healthy People goals, students seemed to perceive these topics as germane and felt competent in these areas. However, most students still wanted more education on public health-related topics and expressed desire to collaborate with other health care professionals. Therefore, educators should consider allocating more time to these topics, with a focus on interprofessional activities and elective courses. This would also align neatly with one of the new developmental objectives of Healthy People 2030, which aims to “increase the inclusion of interprofessional prevention education in the curricula of health professions programs” [15]. This initial study demonstrates many common goals shared by medical and pharmacy students; there are likely similar areas of overlap that could be investigated among other health care professions students. These areas are ripe for cooperative efforts and should be organized more formally to improve the nation’s health. 

This study was subject to several limitations. The results of this study may not be generalizable to all medical and pharmacy students in Ohio due the response rate and lack of participation from five schools of medicine and two schools of pharmacy citing high survey burden among their students. Additionally, while the explanatory email was designed to minimize potential participant bias, there is no information about non-responders, so any potential systematic differences cannot be assessed. While the HPCTF represents eight health professional education associations, only two disciplines were studied here, given the feasibility of identifying and surveying schools offering these varied degrees across Ohio.

Notwithstanding these limitations, this study is significant as it is the first to characterize medical and pharmacy students’ awareness and interest in Healthy People 2030. The results of this study can guide curricular efforts and inform future studies of health professions students. Further research should be conducted to build upon this work and gather data from a larger representative sample of students from all organizational members of the HPCTF to understand their knowledge and interest regarding public health priorities. Additionally, future studies should more fully characterize U.S. health professions students’ perceptions and attitudes regarding public health-related topics in their curricula, as there is scant literature currently [16].

## 7. Conclusions

Although they reported low familiarity with the Healthy People initiative, both medical and pharmacy students in Ohio recognized core public health topics in their curricula and had an interest in elective courses focused on public health topics. Students from both professions identified similar priorities from Healthy People 2030 to impact in future practice. Students also expressed desire to practice interprofessionally to attain public health goals. Further research should involve a more comprehensive sample of health professions students to gain a clearer understanding of the current state and possible improvements for teaching public health-related topics and initiatives. 

## Figures and Tables

**Table 1:** Blueprint for Healthy People 2030 [3].

Vision	A society in which all people can achieve their full potential for health and well-being across the lifespan.
Mission	To promote, strengthen and evaluate the Nation’s efforts to improve the health and well-being of all people.
Foundational Principles	Health and well-being of all people and communities are essential to a thriving, equitable society. Promoting health and well-being and preventing disease are linked efforts that encompass physical, mental, and social health dimensions. Investing to achieve the full potential for health and well-being for all provides valuable benefits to society. Achieving health and well-being requires eliminating health disparities, achieving health equity, and attaining health literacy. Healthy physical, social, and economic environments strengthen the potential to achieve health and well-being. Promoting and achieving health and well-being nationwide is a shared responsibility that is distributed across the national, state, tribal, and community levels, including the public, private, and not-for-profit sectors. Working to attain the full potential for health and well-being of the population is a component of decision-making and policy formulation across all sectors.
Overarching Goals	Attain healthy, thriving lives and well-being, free of preventable disease, disability, injury, and premature death. Eliminate health disparities, achieve health equity, and attain health literacy to improve the health and well-being of all. Create social, physical, and economic environments that promote attaining full potential for health and well-being for all. Promote healthy development, healthy behaviors, and well-being across all life stages. Engage leadership, key constituents, and the public across multiple sectors to take action and design policies that improve the health and well-being of all.
Objectives	355 objectives organized in the following categories: health conditions; health behaviors; populations; settings and systems; social determinants of health

**Table 2:** Selected demographic characteristics of respondents.

Characteristic	No. (%)
Age	(n = 324)
Under 20 years	16 (5)
20–25 years	216 (67)
26–35 years	87 (27)
36–55 years	5 (2)
Anticipated year of graduation	(n = 415)
2021	88 (21)
2022	77 (19)
2023	103 (25)
2024	110 (27)
2025	16 (4)
2026 or later	21 (5)
Self-description of gender	(n = 335)
Male (including transgender men)	100 (30)
Female (including transgender women)	233 (70)
Non-binary	2 (<1)
Currently or previously worked in a medical setting for ≥6 months, including clinical rotations	(n = 336)
Yes	247 (74)
No	89 (26)

Percentages may not total 100 due to rounding.

**Table 3:** Questions used to assess public health in education.

Question	Medical No. (%)	Pharmacy No. (%)
Students’ knowledge of the Healthy People initiative		
Are you familiar with the Healthy People public health initiative? *	(n = 182)	(n = 233)
Yes	31 (17)	92 (39)
No	127 (70)	102 (44)
I’m not sure	24 (13)	39 (17)
What is the mission of the Healthy People initiative?	(n = 182)	(n = 233)
Correct	173 (95)	218 (94)
Incorrect	9 (5)	15 (6)
What is the current edition of the Healthy People? *	(n = 179)	(n = 233)
Correct	41 (23)	77 (33)
Incorrect	138 (77)	156 (67)
Perceived learning in professional curricula		
Have you learned about the Healthy People initiative in your medical [pharmacy] school curriculum? *	(n = 181)	(n = 233)
Yes	13 (7)	84 (36)
No	128 (71)	105 (45)
I’m not sure	40 (22)	44 (19)
My curriculum teaches the foundations of public health (quantitative and analytic skills needed to assess and monitor the health of populations).	(n = 166)	(n = 208)
Strongly agree	33 (20)	59 (28)
Somewhat agree	90 (54)	111 (53)
Neither agree nor disagree	10 (6)	20 (10)
Somewhat disagree	27 (16)	16 (8)
Strongly disagree	6 (4)	2 (1)
My curriculum includes clinical preventive services and health promotion (such as USPSTF guidelines, evidence-based interventions). *	(n = 166)	(n = 208)
Strongly agree	95 (57)	87 (42)
Somewhat agree	64 (39)	85 (41)
Neither agree nor disagree	4 (2)	20 (10)
Somewhat disagree	2 (1)	12 (6)
Strongly disagree	1 (1)	4 (2)
My curriculum integrates clinical practice with population health (individual- and population-based health perspectives). *	(n = 166)	(n = 208)
Strongly agree	45 (27)	86 (41)
Somewhat agree	88 (53)	93 (45)
Neither agree nor disagree	16 (10)	20 (10)
Somewhat disagree	14 (8)	8 (4)
Strongly disagree	3 (2)	1 (<1)
My curriculum explains health systems and health policy (including collaborative efforts between the clinical care and public health communities). *	(n = 166)	(n = 203)
Strongly agree	34 (20)	70 (35)
Somewhat agree	70 (42)	98 (48)
Neither agree nor disagree	19 (11)	25 (12)
Somewhat disagree	35 (21)	8 (4)
Strongly disagree	8 (5)	2 (1)
Which of the following topics have been included in your curriculum? (Select all that apply)	(n = 156)	(n = 203)
Counseling for disease prevention and health promotion	151 (97)	181 (89)
Cultural diversity	145 (93)	179 (88)
Social determinants of health	147 (94)	191 (94)
Evaluation of health sciences literature	107 (66)	156 (77)
Public health systems	88 (56)	154 (76)
Environmental health	59 (38)	78 (38)
Global health	62 (40)	98 (48)
None of these	1 (<1)	2 (<1)
Perceived learning in professional curricula, continued		
I feel knowledgeable about public health related topics.	(n = 162)	(n = 203)
Strongly agree	34 (21)	37 (18)
Somewhat agree	80 (49)	115 (57)
Neither agree nor disagree	27 (17)	27 (13)
Somewhat disagree	18 (11)	21 (10)
Strongly disagree	3 (2)	3 (1)
Desire for additional education		
Please rate your desire to gain more information about public health than your curriculum provides.	(n = 155)	(n = 181)
Strongly interested in more information	72 (46)	69 (38)
Somewhat interested in more information	68 (44)	88 (49)
Not interested in more information	5 (3)	9 (5)
Unsure	10 (6)	15 (8)

Percentages may not total 100 due to rounding. Wording in brackets indicates alternate wording used in the pharmacy student survey. * Statistically significant difference between medical and pharmacy student response (*p* < 0.05).

**Table 4:** Questions used to assess public health in future practice.

Question	Medical No. (%)	Pharmacy No. (%)
Shared public health goals from Healthy People 2030		
I believe public health initiatives are valuable to the American health care system.	(n = 162)	(n = 203)
Strongly agree	144 (89)	166 (82)
Somewhat agree	16 (10)	31 (15)
Neither agree nor disagree	2 (1)	4 (2)
Somewhat disagree	0 (0)	0 (0)
Strongly disagree	0 (0)	2 (1)
Select the following HP 2030 focus area as the most significant to improving the nation’s health. *	(n = 156)	(n = 186)
Health conditions (treating/preventing disease states)	21 (13)	58 (31)
Health behaviors (encouraging behaviors that prevent disease)	47 (30)	62 (33)
Populations (supporting health of specific populations)	2 (1)	3 (2)
Settings and systems (focusing on specific locations/systems)	10 (6)	2 (1)
Social determinants of health (addressing social factors)	76 (49)	61 (33)
Which of the following social determinants of health from HP 2030 do you believe you can provide the most impact in your future clinical practice? (Select up to 5)	(n = 155)	(n = 181)
Economic stability	57 (37)	73 (40)
Education access and quality	81 (52)	117 (65)
Health care access and quality	135 (87)	160 (88)
Neighborhood and built environment	81 (52)	90 (50)
Social and community context	104 (67)	114 (63)
Anticipated barriers		
Do you believe there will be any barriers to providing health promotion and services outlined by public health initiatives, such as Healthy People, in your future practice?	(n = 155)	(n = 182)
Yes	108 (70)	110 (60)
No	4 (3)	10 (5)
Maybe	43 (28)	62 (34)
Interest in interprofessional collaboration		
How important do you believe it is to work with other health care professionals within your profession to achieve these public health goals?	(n = 155)	(n = 181)
Extremely important	125 (81)	152 (84)
Very important	27 (17)	27 (15)
Moderately important	3 (2)	1 (<1)
Slightly important	0 (0)	0 (0)
Not at all important	0 (0)	1 (<1)
How important do you believe it is to work with health care professionals outside of your profession to achieve these public health goals?	(n = 155)	(n = 182)
Extremely important	126 (82)	152 (84)
Very important	28 (18)	27 (15)
Moderately important	1 (<1)	2 (1)
Slightly important	0 (0)	1 (<1)
Not at all important	0 (0)	0 (0)
How do you rate the following statement: “I would be interested in forming collaborative partnerships (collaborate practice agreements) with a pharmacist [physician] to manage certain health conditions in patients”?	(n = 155)	(n = 181)
Strongly agree	127 (82)	152 (84)
Somewhat agree	22 (14)	24 (13)
Neither agree nor disagree	4 (3)	2 (1)
Somewhat disagree	2 (1)	1 (<1)
Strongly disagree	0 (0)	2 (1)
[How do you rate the following statement: “I would be interested in gaining provider status”?]	-	(n = 181)
Strongly agree	-	146 (81)
Somewhat agree	-	27 (15)
Neither agree nor disagree	-	6 (3)
Somewhat disagree	-	1 (<1)
Strongly disagree	-	1 (<1)

Percentages may not total 100 due to rounding. Wording in brackets indicates alternate wording used in the pharmacy student survey. * Statistically significant difference between medical and pharmacy student response (*p* < 0.05).

## Data Availability

The data presented in this study are available on request from the corresponding author.

## References

[1] Tikkanen R., Fields K. The Commonwealth Fund. Multinational Comparisons of Health Systems Data 2020. https://www.commonwealthfund.org/publications/other-publication/2021/feb/multinational-comparisons-health-systems-data-2020.

[2] National Academies of Sciences, Engineering, and Medicine (2017). Communities in Action: Pathways to Health Equity.

[3] Office of Disease Prevention and Health Promotion, U.S. Department of Health and Human Services Healthy People 2030. https://health.gov/healthypeople.

[4] Institute of Medicine (2012). Primary Care and Public Health: Exploring Integration to Improve Population Health.

[5] Centers for Disease Control and Prevention, U.S. Department of Health and Human Services Collaborative Practice Agreements and Pharmacists’ Patient Care Services: A Resource for Pharmacists. https://www.cdc.gov/dhdsp/pubs/docs/translational_tools_pharmacists.pdf.

[6] Choe H.M., Standiford C.J., Brown M.T. Embedding Pharmacists into the Practice. American Medical Association STEPS Forward. www.stepsforward.org/modules/embedded-pharmacists.

[7] Gebhart F. (2019). Provider and Payment Status: An Update. Drug Top..

[8] Gebhart F. (2018). More Pharmacists Move into Medical Practices, More Doctors See Value. Drug Top..

[9] Association for Prevention Teaching and Research APTR Healthy People Curriculum Task Force. https://www.aptrweb.org/page/HPC_Taskforce.

[10] Association for Prevention Teaching and Research Clinical Prevention and Population Health Curriculum Framework. https://www.teachpopulationhealth.org.

[11] Medicaid and Public Health Partnership Learning Series Public Health and Population Health 101. https://www.astho.org/Health-Systems-Transformation/Medicaid-and-Public-Health-Partnerships/Learning-Series/Public-Health-and-Population-Health-101/.

[12] Office of Disease Prevention and Health Promotion, U.S. Department of Health and Human Services Healthy People 2020: Educational and Community-Based Programs. https://www.healthypeople.gov/2020/topics-objectives/topic/educational-and-community-based-programs/objectives.

[13] Borgialli D.A., Camargo C.A. (2010). Healthy People 2010 Emergency Medicine Module: A Multicenter Survey and Educational Intervention. J. Emerg. Med..

[14] Chandra R.N. (2018). Pharmacists’ Knowledge of Social Determinants of Health in Post-Graduate Pharmacy Residency Programs.

[15] Office of Disease Prevention and Health Promotion, U.S. Department of Health and Human Services Healthy People 2030: Schools. https://health.gov/healthypeople/objectives-and-data/browse-objectives/schools.

[16] Mehling K., Jeong S.J. (2018). Perceptions of Public Health: The Challenges of Public Health Education Integration. J. Educ. Dev..

